# A rhythm landscape approach to the developmental dynamics of birdsong

**DOI:** 10.1098/rsif.2015.0802

**Published:** 2015-11-06

**Authors:** Kazutoshi Sasahara, Ofer Tchernichovski, Miki Takahasi, Kenta Suzuki, Kazuo Okanoya

**Affiliations:** 1 Department of Complex Systems Science, Nagoya University, Furo-cho, Chikusa-ku, Nagoya 464-8601, Japan; 2 Laboratory for Biolinguistics, RIKEN Brain Science Institute, 2-1 Hirosawa, Wako-shi, Saitama 351-0198, Japan; 3 Department of Psychology, Hunter College, City University of New York, 695 Park Avenue, New York, NY 10065, USA; 4 Faculty of Health Sciences, Nihon Institute of Medical Science, 1276 Shimogawara, Moroyama-machi, Iruma-gun, Saitama 350-0435, Japan; 5 Department of Life Sciences, University of Tokyo, 3-8-1 Komaba, Meguro-ku, Tokyo 153-8902, Japan

**Keywords:** biological rhythms, birdsong, rhythm landscape, band formation dynamics

## Abstract

Unlike simple biological rhythms, the rhythm of the oscine bird song is a learned time series of diverse sounds that change dynamically during vocal ontogeny. How to quantify rhythm development is one of the most important challenges in behavioural biology. Here, we propose a simple method, called ‘rhythm landscape’, to visualize and quantify how rhythm structure, which is measured as durational patterns of sounds and silences, emerges and changes over development. Applying this method to the development of Bengalese finch songs, we show that the rhythm structure begins with a broadband rhythm that develops into diverse rhythms largely through branching from precursors. Furthermore, an information-theoretic measure, the Jensen–Shannon divergence, was used to characterize the crystallization process of birdsong rhythm, which started with a high rate of rhythm change and progressed to a stage of slow refinement. This simple method provides a useful description of rhythm development, thereby helping to reveal key temporal constraints on complex biological rhythms.

## 1. Introduction

Chronobiological studies have shown that biological systems exhibit various rhythmic behaviours on a wide range of temporal and spatial scales at both the individual and collective level, including cell cycles, circadian rhythms, synchronous signals and collective motions [1]. These behaviours are often periodic or monotonic rhythms based on genetics or innate ability. By contrast, the oscine birdsong is a sequence of diverse sounds with rhythmic patterns that can drastically change throughout developmental learning with innate guidance. Early in life, juveniles internalize adult songs to create a ‘song model’ and then gradually match their own song to this model via sensory–motor coordination [2]. Birdsong can, therefore, serve as a model system that allows us to investigate complex biological rhythms beyond the level of simple biological clocks and innate rhythm generators.

Early pioneering studies of birdsong development were mostly based on visually examining sound spectrograms and addressing clearly recognizable developmental traits [3,4]. Recent improvements in recording techniques have enabled us to trace the entire developmental process in more detail [5,6], shedding light on unexplored time-dependent behavioural changes, such as the song imitation process [7], the stepwise learning of pairwise transitions [8] and the evolution of song culture [9]. Many previous studies on rhythm development were carried out using the simple stereotypic song of zebra finch [5,10] and some of the neural and behavioural mechanisms related to rhythm generation have been reported [11–14].

Here, we focused on the song of Bengalese finch (*Lonchura striata* var. *domestica*) as a model of complex biological rhythms. The Bengalese finch song is a sequence of a variety of note types, some of which form substring patterns (referred to as ‘chunks’) [15,16]. The set of sequencing rules for these notes and chunks (referred to as ‘song syntax’) is flexible, and may be modelled using various computational methods [17–19]. The structural complexity of the Bengalese finch song is highly variable across individuals, ranging from a linear stereotypic sequence to a branched variegated sequence; nevertheless, it is, in general, more complex and flexible than the zebra finch song. This study represents the first attempt to quantify rhythm development in the more complex Bengalese finch song. The findings presented here should therefore be beneficial not only for making a comparison with simpler birdsongs but also for obtaining novel insights into rhythm development.

What is biological rhythm? The definition of rhythm varies markedly between disciplines and is often considered from the psychological aspects of sound perception. However, we instead focus on the acoustic aspects of sound production by quantifying durational patterns that consist of sounds and silences, which represents one of the simplest definitions of rhythm [20]. To quantify such durational patterns, we specifically measure the inter-onset intervals (IOIs) of two adjacent onsets of notes, and consequently the overall distribution of IOIs corresponds to rhythm structure. In other words, we focus on the accuracy of onset control in note sequencing, using the IOI distribution as a proxy for birdsong rhythm. Such an approach is often used in music signal processing [20] but is rarely applied to biological rhythms, especially rhythm development. Therefore, the primary objective of this study was to establish a simple method for visualizing and quantifying rhythm development that can be applied to a large amount of birdsong data (ranging from juveniles to adults) in a comparable manner. The secondary objective was to validate this method experimentally by demonstrating the developmental dynamics of complex Bengalese finch songs.

## 2. Material and methods

### 2.1. Birdsong data

We used 12 male juvenile Bengalese finches from eight different colonies at the Laboratory for Biolinguistics, RIKEN Brain Science Institute (BSI). To trace the entire process of rhythm development, a 24 h recording was conducted for each juvenile about every 4 days starting at about day 40 after hatching. During a recording, a juvenile was kept in a soundproof box equipped with a microphone (Audio-Technica AT3031) and Sound Analysis Pro (an open source software, publicly available at http://soundanalysispro.com) [5], and songs were automatically recorded into wav files (16-bit, mono, 44.1 kHz sampling rate) with serial recording IDs. During the rest of the recording, the juvenile was housed with his father and siblings, during which time he was exposed to conspecific songs from adult male birds (mostly from his father), learning individually distinct songs. Therefore, our recording data are intermittent snapshots of song development under a semi-natural environment. All recordings were conducted in accordance with the guidelines of RIKEN BSI.

Preprocessing for noise reduction was carried out using a bandpass filter (bandwidth 1–9 kHz), and automatic gain control was applied to all recordings using GoldWave (GoldWave Inc.). For each bird, notes were detected from all of the preprocessed data, and two temporal features (i.e. note onset and note duration) were recorded with the time stamp using the batch processing application from Sound Analysis Pro. These temporal features were then used to quantify rhythm, as described below. The dataset is publicly available (http://dx.doi.org/10.7910/DVN/42V7E0). We also computed sound spectrograms (9.27 ms fast Fourier transform data window, 0.5 ms advancing window) for all birds to observe their vocal development.

### 2.2. Rhythm landscape approach

#### 2.2.1. Visualization of the rhythm landscape

We computed IOIs by measuring the temporal differences between the onset positions of consecutive notes *s_i_* and *s_i_*_+1_, defined by IOI(*i*) = *t*(*s_i_*_+1_) − *t*(*s_i_*), and used the IOI distribution as a proxy for the first-order rhythm structure, which is based on note-to-note transitions (note that higher order rhythm structures can be characterized by transitions of two consecutive notes or more). According to sound spectrograms of the recorded data, we found that sounds less than 15 ms and greater than 350 ms were mostly noise, and thus eliminated these data to improve the accuracy of the measurement of IOIs. Each wav file, as mentioned above, has a serial recording ID assigned in the recorded order (i.e. developmental order). For each bird, IOIs were measured file-by-file in the recorded order, resulting in a list of pairs of recording ID and IOI values. From these data, we constructed a two-dimensional histogram of the recording IDs and the IOIs with 300 bins for the two dimensions, and then normalized by dividing by the total number of data in order to convert the probability distributions, which we refer to as ‘rhythm landscape’. The rhythm landscape is visualized using a pseudo colour plot, as shown later. Note that the above bin setting was used only for the sake of visualization.

#### 2.2.2. Quantification of the rhythm landscape

To quantify the rhythm landscape, we constructed the histogram of IOIs for each recording day, using a range between 0 and 350 ms with 10 ms bins (i.e. index *i* ranges from 1 to 35), and normalized by dividing by the data size, which we refer to as the ‘IOI distribution’. Then, we smoothed the IOI distribution by a kernel density estimate with a range between 0 and 350 ms to estimate the local maxima, which were deemed as ‘bands’. Scipy (http://scipy.org/scipylib/), a signal processing library, was used for the smoothing as well as the estimation of band positions and those numbers.

To measure the rhythm proficiency (i.e. the degree of completion in rhythm development), we compared the IOI distributions for each recording day using an information-theoretic measure, called the Jensen–Shannon divergence (JS) [21]. This is a symmetric version of the Kullback–Leibler divergence (KL) that quantifies how close a probability distribution *Q* = {*q_i_*} is to a model probability distribution *P* = {*p_i_*}. JS is always well defined and bounded and thus suitable for biological data:2.1
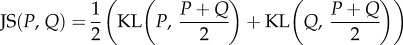
and2.2
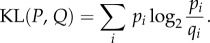


In the following analysis, *P* was the IOI distribution for the final recording day that was determined based on sound spectrograms of the recorded songs and *Q* was that for each of the other recording days. By doing so, the JS works as a dissimilarity measure: the JS value approaches zero as the developmental rhythm (*Q*) becomes more similar to the crystallized adult rhythm (*P*).

The greatest advantage of the rhythm landscape approach is its simplicity. The measurement of IOIs can be applied to undeveloped juvenile songs as well as crystallized adult songs in the same manner, so rhythm development is easily tractable. Furthermore, the results can be intuitively understood because the IOI value corresponds to a note-to-note transition.

## 3. Results

Our analyses first addressed macro-level patterns, followed by micro-level changes in the rhythm landscape. The macro- and micro-level analyses were then interpolated by examining song development at the substring level.

### 3.1. Branching trajectories in a rhythm landscape: an example

**Figure 1.**
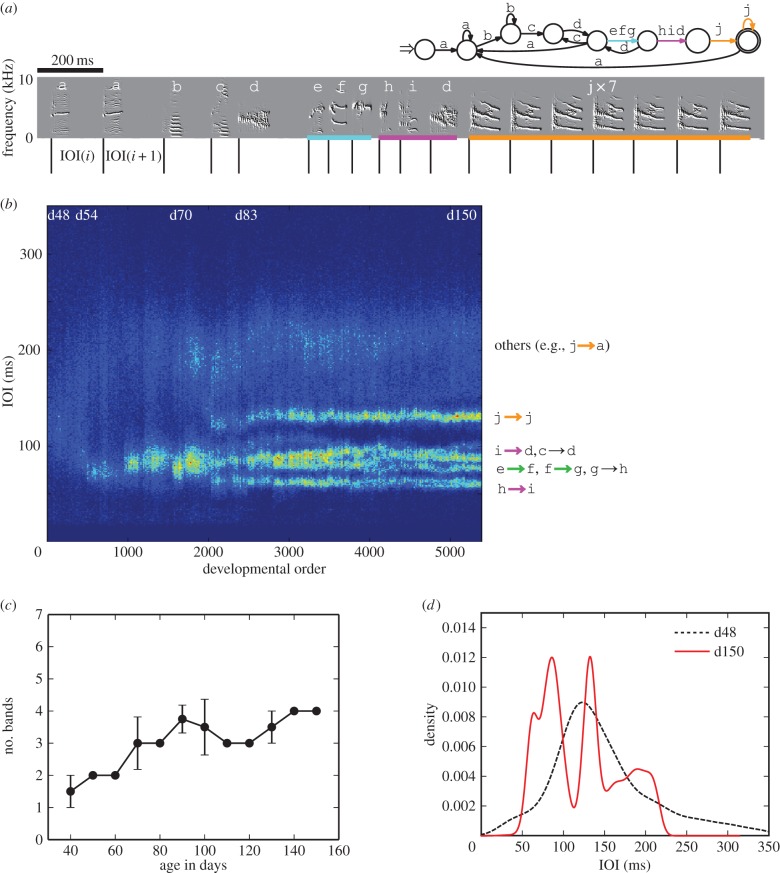
Bengalese finch song and its rhythm landscape (bird1). (*a*) A variegated song and its song syntax (transition diagram) constructed as previously described [17] from 30 songs in the last recording. Letters denote note types and colours correspond to different chunks. Black vertical lines denote note onsets and intermediate spaces denote IOIs. (*b*) Two-dimensional visualization of the rhythm landscape, where the brighter points denote higher density (*N* = 521137). (*c*) Development of the number of bands in (*b*) (mean ± s.d.). (*d*) IOI distributions for day 48 and 150 after hatching.

To demonstrate how the rhythm landscape approach works, we begin with an example of a single bird (bird1) whose song includes many note types and diverse transitions among notes (figure 1*a*). In the rhythm landscape for bird1 (figure 1*b*), we see developmental trajectories, implying that a broadband rhythm had developed into diverse rhythms by gradually increasing the number of bands, as shown in figure 1*c*. For instance, the bands of less than 100 ms gradually arose by branching, whereas the band of 130 ms seems to be a sudden emergence without precursors (see also the electronic supplementary material, figure S1). The clear bands at the endpoint were associated with specific note-to-note transitions: the bands of less than 100 ms were mainly related to transitions such as h → i and i → d, while the 130 ms band was specifically related to the self-repetition of j → j. By contrast, the upmost trajectory was not clear because the associated transitions (e.g. a → a and j → a) were less frequent or more variable, or both. In figure 1*d*, a comparison of the IOI distributions between day 48 and 150 shows that there was greater fluctuation in the initial rhythm and that new stable temporal patterns emerged during development. It should be noted that the rhythm landscape does not discriminate every single transition but emphasizes stable dominant transitions, which are the key ingredients of rhythm.

As Bengalese finches learn an individually distinct song depending on the acoustic environment in which they are raised, it is natural that branching trajectories in the rhythm landscape differ among individuals, as seen in figure 1*b* and the electronic supplementary material, figure S2*b*. The rhythm landscapes for other birds (*n* = 10) are shown in the electronic supplementary material, figures S4–S8.

### 3.2. Common properties of rhythm development

**Figure 2.**
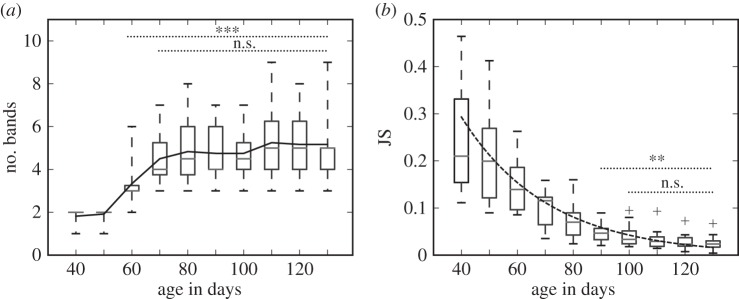
Common properties of rhythm landscapes. (*a*) Boxplots of the number of bands averaged per individual by 10 days from day 40 after hatching. (*b*) Box plots of JS values (i.e. dissimilarity to the crystallized song) averaged per individual by 10 days from day 40 after hatching, with the best exponential fit. The significances refer to differences between day 130 and before that.

We examined common properties of the band formation patterns and processes across all birds during rhythm development. For this, we conducted the band analysis of the smoothed IOI distributions for each recording day, as described in §2.2.2. The boxplots in figure 2*a* show that the number of bands increased with development, starting from one or two bands and reaching an average of about five by day 70 without significant change thereafter (paired *t*-test, *n* = 12, *p* > 0.01, n.s.). Furthermore, we characterized how rhythm proficiency progressed with development by measuring the JS divergence, used as a dissimilarity measure between juvenile and adult rhythms. Figure 2*b* shows that the mean JS values approximately followed an exponential curve (*y* = 0.97e^−0.03*x*^, *χ*^2^ residual = 0.0006), indicating that the juvenile rhythm developed quickly before day 90 and then crystallized, after which the IOI distributions were not statistically different (paired *t*-test, *n* = 12, *p* > 0.01, n.s.). A comparison of the fitted exponent with other species would be informative, because it corresponds to the speed of rhythm development. These results indicate that the number of bands reached its maximum by day 70, where the birds had all IOI elements but still required around one month to crystallize their rhythms.

As it is likely that birds with more bands may have difficulty in improving the accuracy of note onset control, we addressed individual differences in rhythm development by examining the relationship between the number of bands and the final value of JS across all birds. We found no significant negative correlation between the number of bands and the final value of JS, which was also the case for the number of transition types and the number of note types (Pearson's product–moment correlation, *n* = 12, *p* > 0.01, n.s.).

Next, we conducted a band analysis to identify band formation processes on the rhythm landscape during early development. We limited our analysis to band emergence events that occurred before day 80. The band analysis followed two steps. First, we looked into the emergence of new bands from the stem band on the rhythm landscape and classified these events as either an event that exhibited a clear continuous branching (‘branching’) or an event that exhibited no clear continuous branching (‘other’). We focused on reliable branching events because our data are intermittent recordings conducted about every 4 days, thus making emergence events difficult to determine (e.g. if we find a discontinuous band seemingly formed by sudden emergence without precursors, we cannot rule out the possibility that the band arose from branching that occurred between recordings). Second, we estimated the positions of the stem band and the number of band emergence events by computing the local maxima of the smoothed IOI distribution for each recording day, as described in §2.2.2. Here, the stem band was defined as the local maximum of the smoothed IOI distribution at the recording day just before the emergence of a new band. If there are two local maxima, the one closest to the new band was considered to be the stem band.

**Figure 3.**
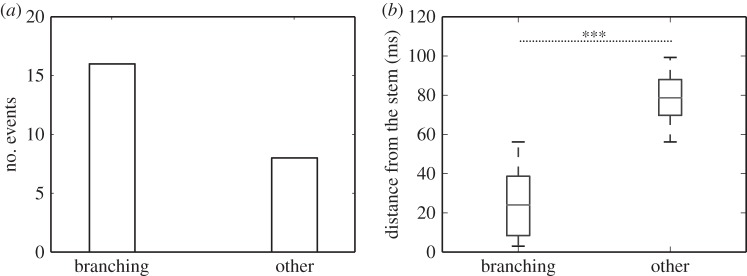
Characteristics of band formation. (*a*) Numbers of bands created by branching and other events before day 80. (*b*) Distance of new bands from the stem before day 80.

Figure 3*a* shows that many of the identified bands surely appeared via branching from precursors before day 80. Indeed, branching events were about twice as common as other events. Interestingly, there was no significant difference in the first day at which the new bands emerged (branching: day 60 ± 4, other: day 62 ± 7; *t*-test, *n* = 24, *p* > 0.01, n.s.); however, there was a significant difference in the distance between the new bands at the first appearance and the stem bands (branching: 24.6 ± 18.7 ms, other: 78.3 ± 15.6 ms; *t*-test, *n* = 24, *p* < 0.001). These results suggest the existence of developmental constraints on band formation: when generating a new temporal pattern, similar but somewhat different temporal patterns can emerge by branching with the same neural and articulatory mechanisms; whereas it might not be reasonable to use branching to obtain new, distant temporal patterns by modifying preexisting patterns.

### 3.3. Song changes at the substring level: two examples

**Figure 4.**
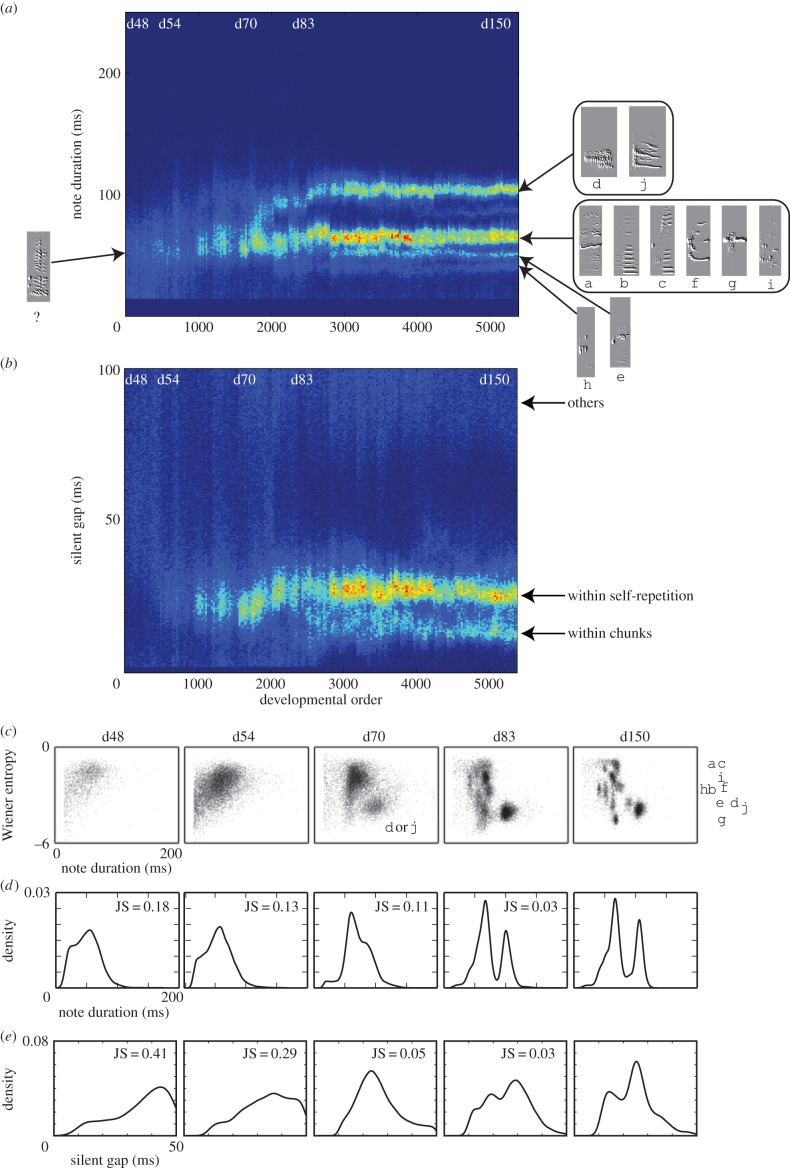
Song changes at the substring level (bird1). Two-dimensional visualization of developmental landscape for (*a*) note duration (*N* = 540227) and (*b*) silent gaps (*N* = 480561). (*c*) Scatter plots of Wiener entropy against note duration, showing the differentiation of 10 note types from a primitive vocalization. Distribution of (*d*) note duration and (*e*) silent gaps at different days.

We show examples of song changes related to band formation during rhythm development. Considering again bird1 in figure 1, in order to observe song changes at the substring level, we constructed developmental landscapes for note duration and silent gaps separately in the same manner as done for the rhythm landscape. In figure 4*a*, a clear branching in note duration was observed between day 70 and 83, during which notes with a longer duration were differentiated from others. Similarly, in figure 4*b*, the silent gaps differentiated into two distinct durations after day 83; the bands of less than 25 ms were associated with j → j and transitions within chunks (e.g. efg and hid), while the other broad band was related to gaps between song bouts. Another example of bird2 is shown in the electronic supplementary material, figure S3*a*,*b*. These examples show that the changes at the substring level, especially the emergence of note types, play a critical role in shaping the rhythm landscape in the Bengalese finch song.

We further investigated song changes at the substring level for each recording day in bird1. Figure 4*c* shows the developmental changes of notes in an acoustic feature space that consists of Wiener entropy (the width and uniformity of the power spectrum) [5] and note duration, in which each dot represents a note and different clusters correspond to different note types. In addition, we show the histograms of note duration and gap duration for each recording day in figure 4*d* and *e*, respectively. A comparison among figure 4*c*–*e* suggests that the band formation was associated with differentiation from undeveloped sounds to a variety of note types: the notes d and j stemmed from the primitive vocalizations at day 70; then new notes such as e and h were further differentiated; 10 note types emerged by day 150 (also see DVDmap.mov in the electronic supplementary material). As shown in this example, the development of note types in Bengalese finches is similar to that of zebra finches [5] but different in that the Bengalese finches have more clusters (i.e. more note types) with blur boundaries. The blur boundaries reflect the fact that in the Bengalese finch song, the same note can appear in different sequential context and has slightly different acoustic features depending on its position in that context. Another example of bird2 is shown in the electronic supplementary material, figure S3*c*–*e*.

**Figure 5.**
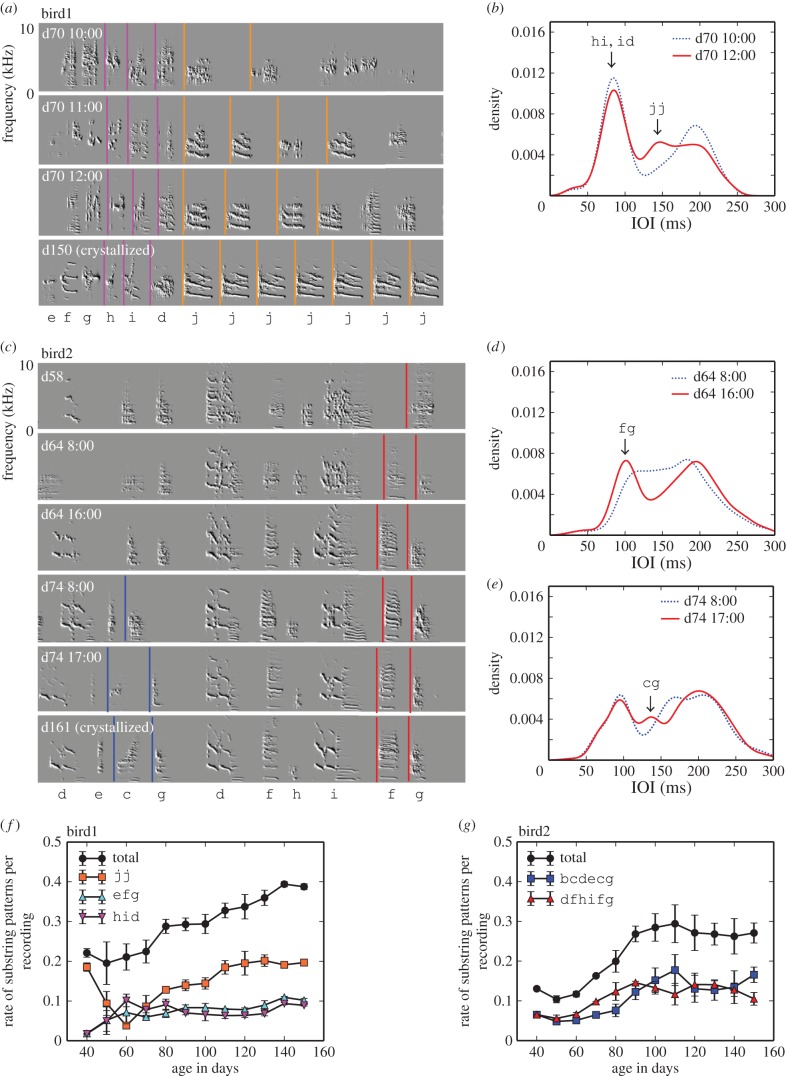
Development of substring patterns. (*a*) Sound spectrograms of developmental songs and (*b*) hourly changes of IOI distribution in bird1 (*N* = 6740 for 10 : 00, *N* = 3309 for 12 : 00). (*c*) Sound spectrograms of developmental songs and (*d*,*e*) hourly changes of IOI distributions in bird2 at day 64 (*N* = 4875 for 8 : 00, *N* = 1300 for 16 : 00) and day 74 (*N* = 3031 for 8 : 00, *N* = 3182 for 17 : 00). In (*a*) and (*c*), letters denote note types, coloured vertical lines correspond to the IOIs for particular transitions with self-repetitions or chunks. Use rate of substring patterns per recording at different days for bird1 (*f*) and bird2 (*g*) (mean ± s.d.).

Juveniles often exhibit rapid song changes at the substring level, which can instantly reshape the rhythm landscape. Here we highlight two examples. According to figure 5*a*,*b*, bird1 improved its repetition skill of j within 2 h, during which bimodal peaks changed into trimodal peaks in the IOI distribution (JS(10 : 00, 12 : 00) = 0.018), and finally the transition j → j became more repeatable ranging from 1 to 10 (the median was 6) at day 150. Note that at day 70, the hid chunk was almost matured in time but not in acoustics (figure 5*a*). Bird2 shows another example of the rapid development of chunks within a day. In figure 5*c*,*d*, the frequency of f→g increased within 8 h at day 64 (JS(8 : 00, 16 : 00) = 0.014) by increasing g at the tail of the preexisting chunk dfhif. Similarly, the frequency of c → g increased within a 9 h period at day 74 (JS(8 : 00, 17 : 00) = 0.025). The rapid onset of chunks and self-repetitions were observed in all birds before day 90, suggesting that rhythm development is not only a result of a slow physical maturation of the nerve system and vocal organ but also that of a fast, trial-and-error learning process.

To examine whether these substring patterns per recording increase with development in these birds, we traced the frequencies of IOIs for these developed chunks and self-repetitions (e.g. jj is 132 ± 4 ms and hid is 160 ± 4 ms) back to the early development. These IOI durations were used as the stand-ins for the chunk-types jj and hid. Notice that the IOI for hid is the temporal difference between the onset positions of h and d. Figure 5*f*,*g* shows that the use rate of these chunks and self-repetitions increased with development, indicating that the accuracy of these substring patterns gradually improved towards a crystallized adult rhythm.

### 3.4. Link between rhythm landscape and song properties at the substring level

**Figure 6.**
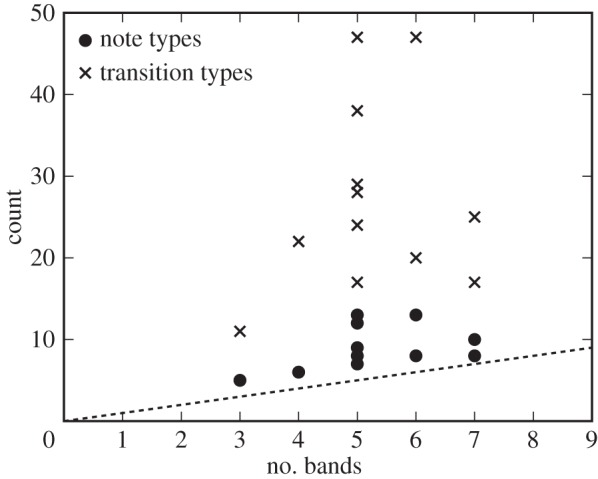
Relationship between IOI bands and song properties. The number of transition types and note types that are above the reference line, suggesting that a large variety of note types and transition types shared the same IOI bands.

As we have seen thus far, the clear band formation in the rhythm landscape reflects a process in which a variety of note types emerge and develop over time, becoming accurately sequenced with specific silent gaps, often associated with chunks and self-repetitions. Finally, we address the question of how clear bands link with the song properties at the substring level. For all birds, we estimated the number of note types and transitions among them by examining sound spectrograms of 30 songs selected randomly from the final recording. Figure 6 is the estimated results as a function of the number of bands, showing two key features. First, the number of different transitions was above the reference line that is in proportion to the number of bands, suggesting that a large variety of transitions belonged to a relatively small number of bands. Second, contrary to our expectation, the number of bands had no correlation with the number of different transitions (Pearson's product–moment correlation, *r* = 0.32, *n* = 12, *p* > 0.01, n.s.), but did have a significant positive correlation with the number of note types (Pearson's product–moment correlation, *r* = 0.69, *n* = 12, *p* = 0.0029). This result evidenced that a growing diversity of note types played a key role in generating new rhythms in Bengalese finch songs. Taken together, the fact that different transitions largely shared the same IOI durations leads us speculate that the same rhythm generator in the brain might be used for different transitions, which should be tested in the future by recording the propagation patterns of burst spikes in HVC neurons [22].

## 4. Discussion

We have proposed the use of a rhythm landscape that takes advantage of the IOI distribution as a proxy for the first-order rhythm structure, in which rhythm development is depicted as a band formation dynamics. Using this approach to the development of Bengalese finch songs in a semi-natural environment, we demonstrated that rhythm development exhibits branching and emerging trajectories along which primitive vocalizations developed into diverse note types followed by specific silent gaps, with accurate and increasing use of substring patterns. While the trajectory patterns considerably differed among birds, the rhythm proficiency progressed exponentially in all birds, beginning with a high rate of rhythm change that was sustained until day 90, followed by a stage of slow refinement, which was independent of the number of transition types and the number of note types in songs. Interestingly, the formation of chunks and self-repetitions, which are important elements of adult rhythm, often exhibited a rapid onset within a single day (or less). The generative process of bands that we observed implies key temporal constraints on rhythm development; that is, the same neural and articulatory mechanisms could be employed for the branching of preexisting rhythms to generate new but similar temporal patterns; however, those mechanisms should not be used to generate new, distant temporal patterns. Furthermore, the observed band formation patterns cannot be explained simply as local production epiphenomena caused by physiological constraints such as co-articulation effect (e.g. notes with longer durations tend to be followed by short gaps for breathing [23]). These results suggest that rhythm development in birdsong is not just a reflection of a slowly maturing central nerve system and vocal organ but also of a result of fast, trial-and-error learning across different levels of song organization. As demonstrated, although the development of birdsong rhythm is dynamic, this can be concisely understood in terms of onset control by projecting onto the time domain using the rhythm landscape.

Several studies of the zebra finch song have reported the differentiation of syllable types [7] and broad unimodal distributions of syllables and silent gaps during early development [12]. Here, we describe equivalent processes in the more complex Bengalese finch song. The Bengalese finch song, however, exhibited more clusters (i.e. more note types) with blur boundaries in note development, and the branching dynamics of rhythm was more diverse; although the same approach should be applied to the zebra finch song for a direct comparison. Furthermore, we previously reported that after 60–80 days of age, juveniles increased transition diversity by acquiring branching transitions (e.g. a → b and a → c) and bidirectional transitions (e.g. a → b and b → a) in a stepwise manner, starting from a more restricted set of transitions [8]. Because the rhythm landscape captures a gradual process of song syntax development in the time domain, including early developmental stages where note types cannot be clearly identified, our results of branching trajectories support and generalize the above finding.

Based on these findings, we propose several future directions for this study. In the early developmental stages, we observed that all possible notes were rapidly emerging from undifferentiated sounds, developing not only their acoustics but also pairwise transitions. This raises the question of how these different levels of song organization relate to each other before the stepwise development of song syntax and rhythm. To address this hierarchical interaction, sparsely sampled data such as those presented here are inadequate and continuous recordings from a very early developmental stage with many individuals would be required. Furthermore, although researchers have proposed some novel analytical methods for birdsong structure [17–19,24–26], both the theories and applications of behavioural sequence data remain open to expansion.

The method described here has both advantages and disadvantages compared to other approaches. While note duration histograms and rhythm landscapes contain more or less the same information if silent gaps vary on a small scale during song development, these diverge in cases of songs with non-constant gaps. Therefore, we would expect that applying this method to complex songs with more variable gaps could better describe the developmental dynamics of such complex songs. Moreover, our method is simple, requiring only the onset information and without the need to compute other features, such as note duration, offset, pitch or frequency modulation. Thus, the rhythm landscape provides a new option and functions as a first stage estimator before applying a more detailed and computationally costly method. We would be able to compute the rhythm landscape much faster by developing a specialized algorithm for detecting note onsets, which is also a promising future direction. However, this approach is potentially disadvantageous for the study of song changes at the subnote level or for gaining neuro-mechanistic insights into the development of notes and silent gaps, which are known to be governed by different neural mechanisms [12]. In such cases, the rhythm landscape might be a little too ‘coarse-grained’, because it does not describe every single transition but rather emphasizes stable major transitions with fewer bands. Although there are alternative approaches, e.g. the rhythm spectrogram is one suited to the continuous recording of simple zebra finch songs [10], this method would not be suitable for capturing intermittent recordings of song development, such as in our data. In such a case, the rhythm landscape is more suited to attempting to obtain an overarching picture of birdsong development.

The rhythm landscape is reminiscent of the Waddington epigenetic landscape, a conceptual metaphor for cell development [27]. The original epigenetic landscape is static and metaphorically represents potentials genetically determined at birth, and it lacks physical units and dynamics. By contrast, the rhythm landscape is a learned landscape, reflecting a combination of biological and cultural processes. From this landscape, we can obtain quantitative knowledge about the dynamic process by which rhythmic patterns emerge, diverge and canalize (stabilize) in the course of vocal ontogeny (electronic supplementary material, figure S9). Therefore, we conclude that the rhythm landscape is a useful approach to explore the dynamics of complex biological rhythms, including biological clocks and rhythm generators, from a large amount of developmental data.

## Supplementary Material

Rhythm landscapes of the Bengalese finch
