# Supplementary material for: A rhythm landscape approach to the developmental dynamics of birdsong
Source: J R Soc Interface. 2015 Nov 6;12(112):20150802. doi: 10.1098/rsif.2015.0802 (PMC4685852; doi:10.1098/rsif.2015.0802)
Supplement: Rhythm landscapes of the Bengalese finch [file rsif20150802supp1.pdf]

Electronic supplementary material for
A rhythm landscape approach to the developmental dynamics of birdsong rhythm

Kazutoshi Sasahara, Ofer Tchernichovski, Miki Takahasi,
Kenta Suzuki, and Kazuo Okanoya

To whom correspondence should be addressed:
sasahara@nagoya-u.jp

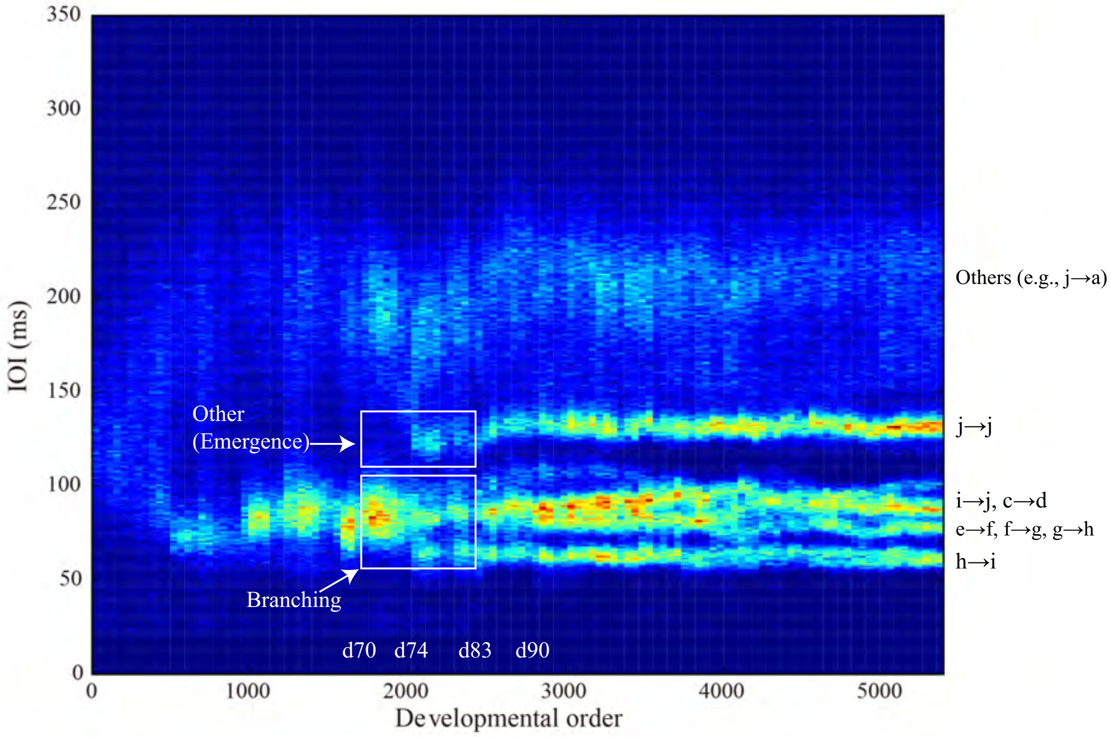

Figure S 1: **Rhythm landscape of bird1 (a higher resolution version of Fig. 2B).**

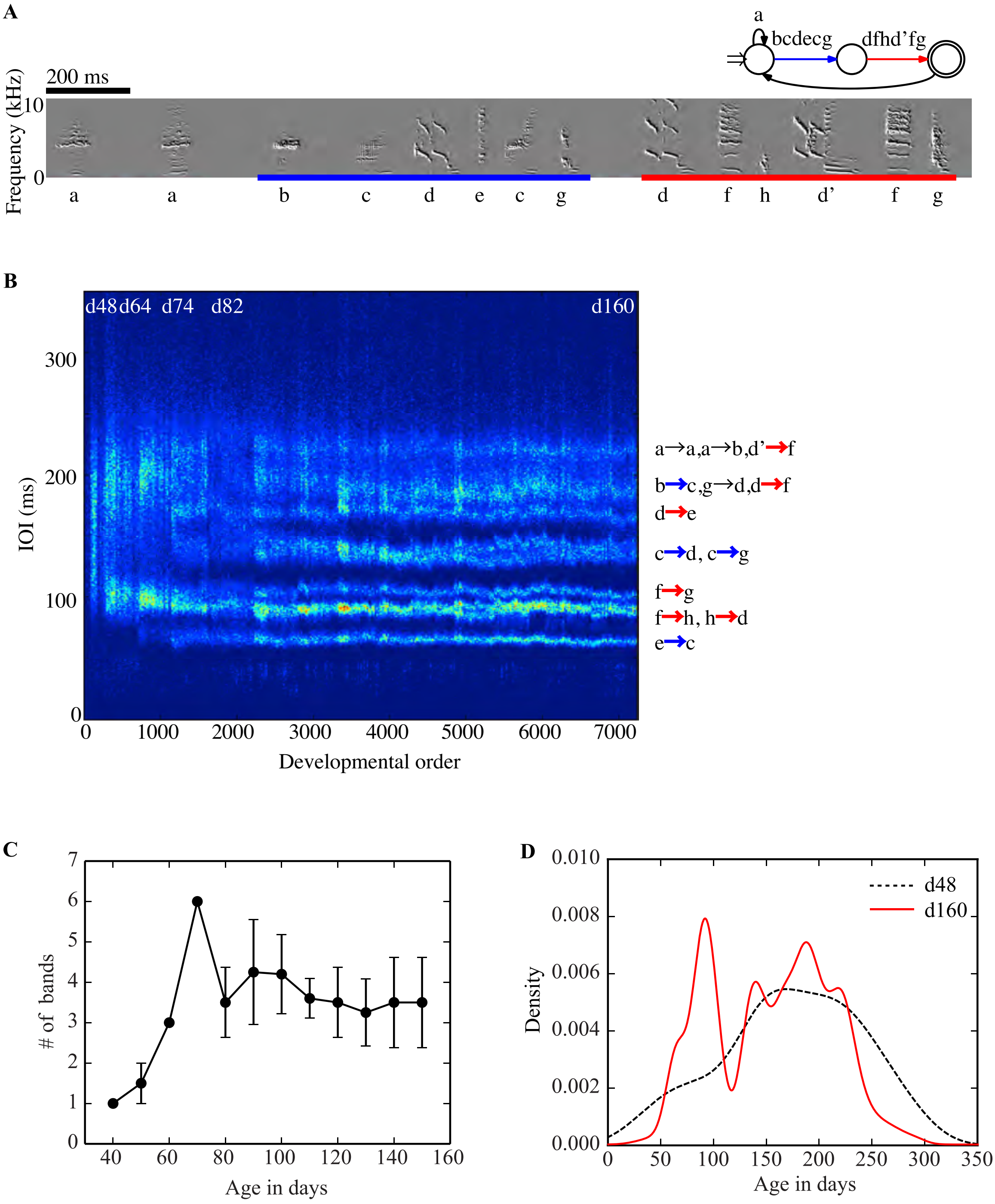

Figure S 2: **Bengalese finch song and its rhythm landscape (bird2).** (A) A stereotypic song and its song syntax (transition diagram) constructed as previously described [1] from 30 songs in the last recording. Letters denote note types and colors correspond to different chunks. Black vertical lines denote note onsets and intermediate spaces denote inter-onset intervals (IOIs). (B) 2D visualization of the rhythm landscape where the brighter points denote higher density (N=484786). (C) Development of the number of bands in (B) (mean ± sd). (D) IOI distributions for day 48 and day 160 after hatching.

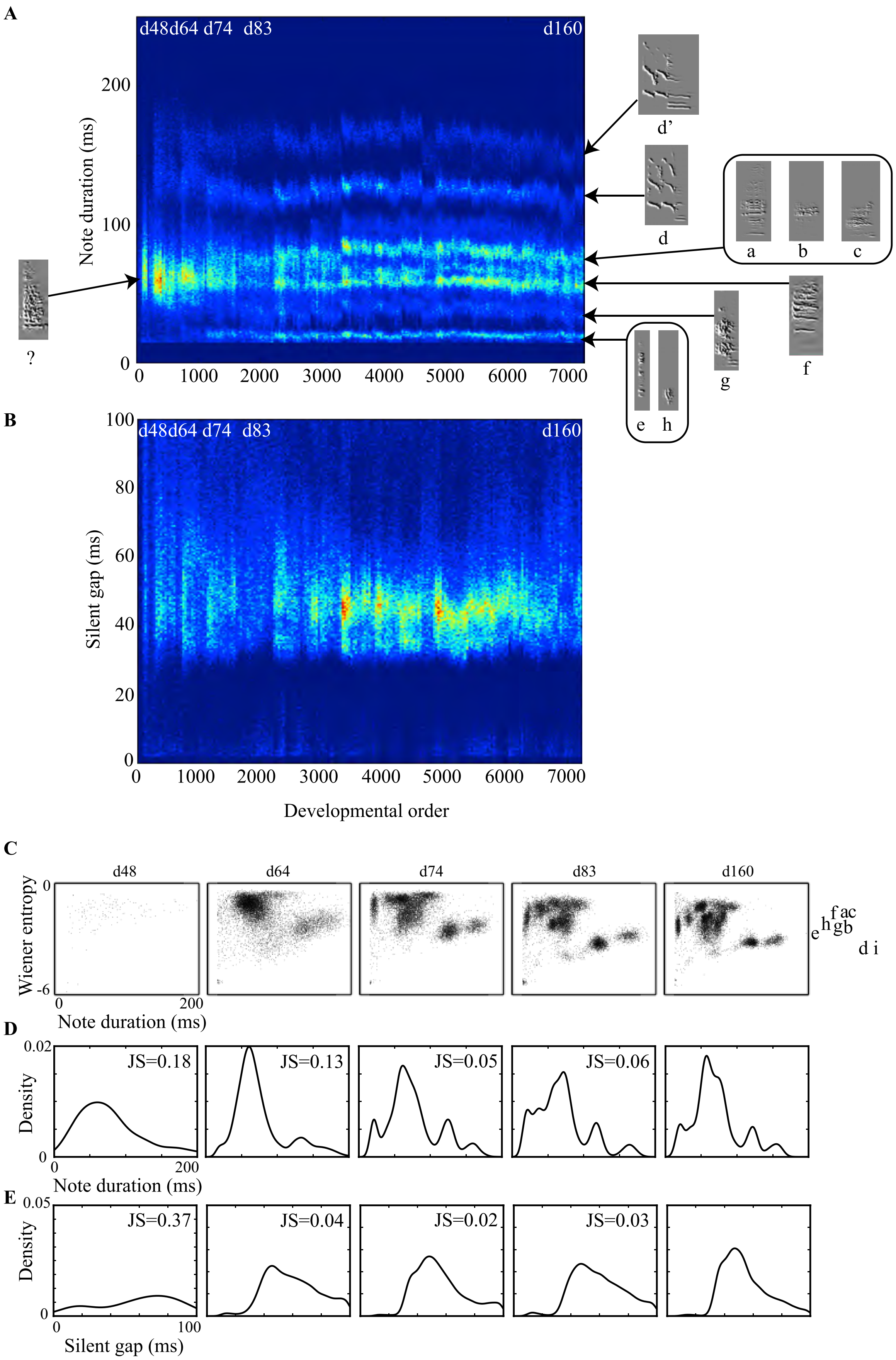

Figure S 3: **Song changes at the substring level (bird2).** 2D visualization of developmental landscape for (A) note duration (N=534499) and (B) silent gaps (N=485280). (C) Scatter plots of Wiener entropy against note duration, showing the differentiation of 10 note types from a primitive vocalization. Distribution of (D) note duration and (E) silent gaps at different days.

A

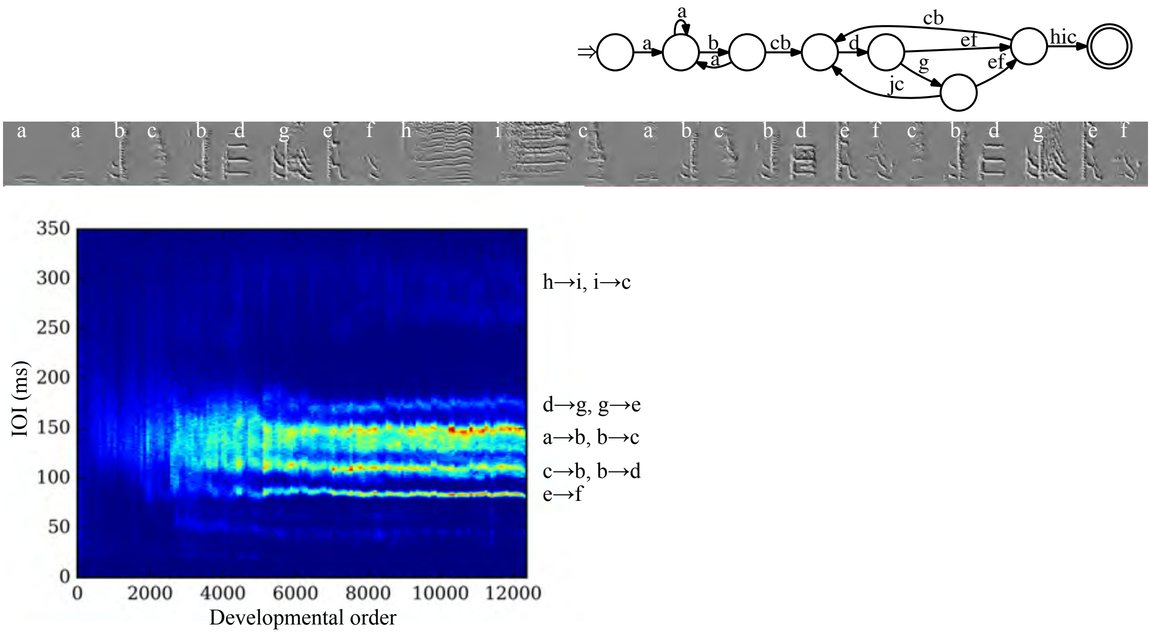

B

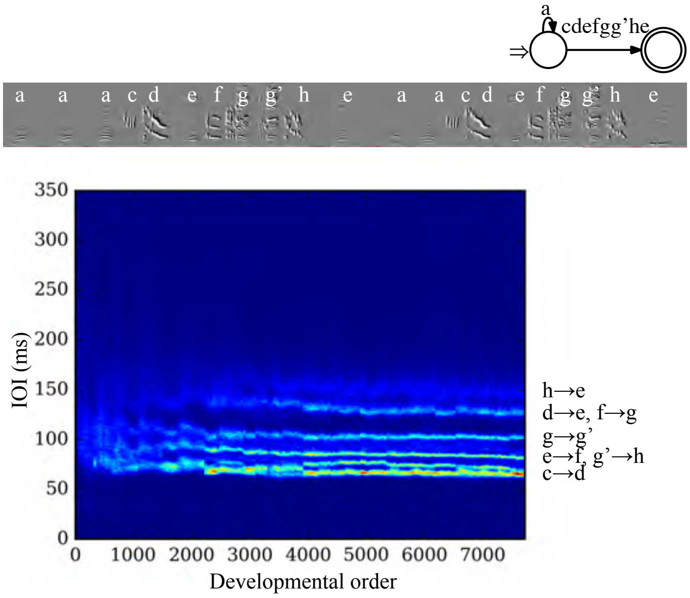

Figure S 4: **Examples of rhythm landscape.** (A) Bird3: # of note types = 10, # of transitions = 25. (B) Bird4: # of note types = 8, # of transitions = 17. Major transitions and transition diagrams are shown.

C

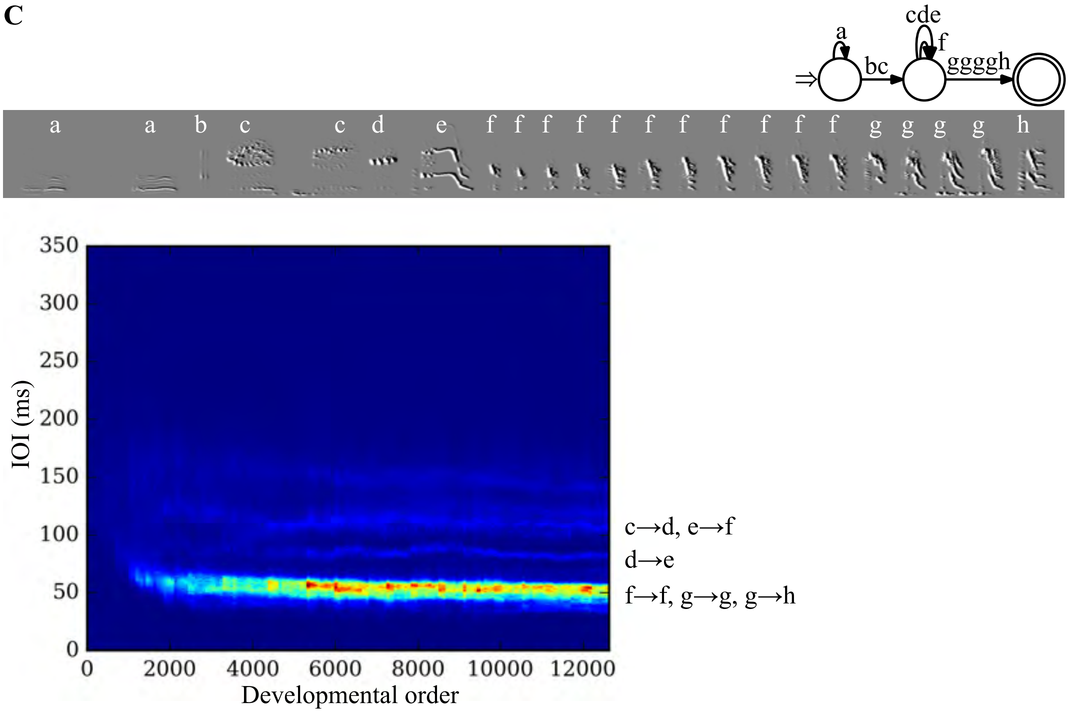

D

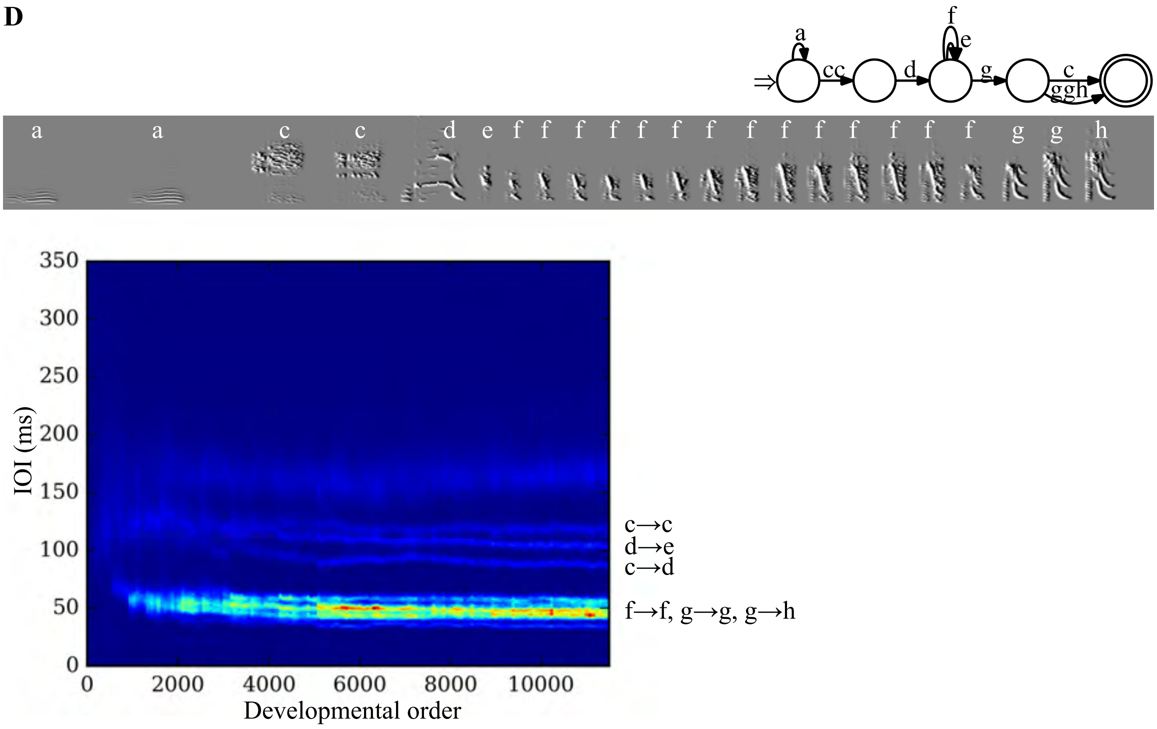

Figure S 5: **Examples of rhythm landscape.** (C) Bird5: # of note types = 8, # of transitions = 24. (D) Bird6: # of note types = 7, # of transitions = 28. Major transitions and transition diagrams are shown.

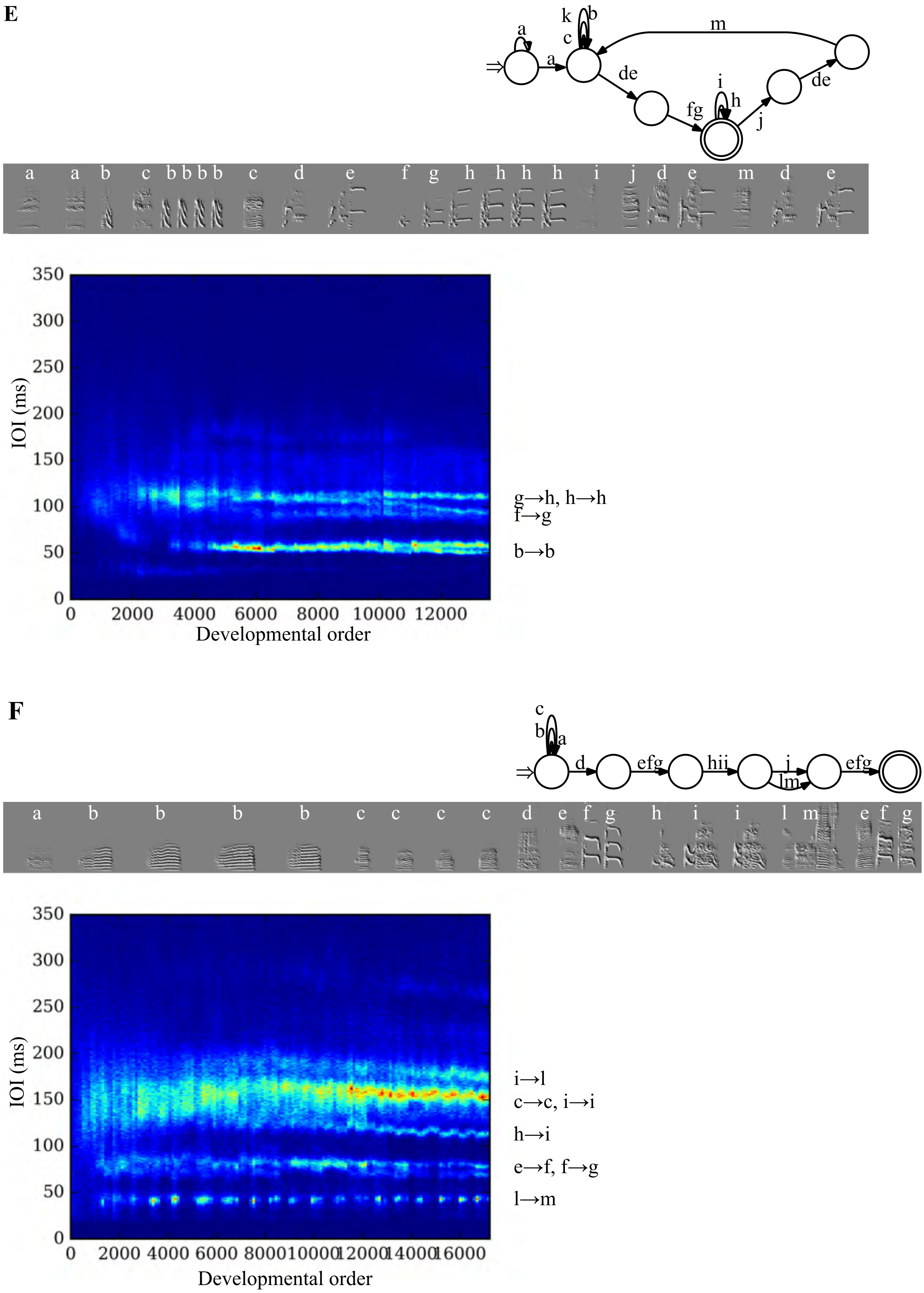

Figure S 6: **Examples of rhythm landscape.** (E) Bird7: # of note types = 13, # of transitions = 47. (F) Bird8: # of note types = 13, # of transitions = 47. Major transitions and transition diagrams are shown.

G

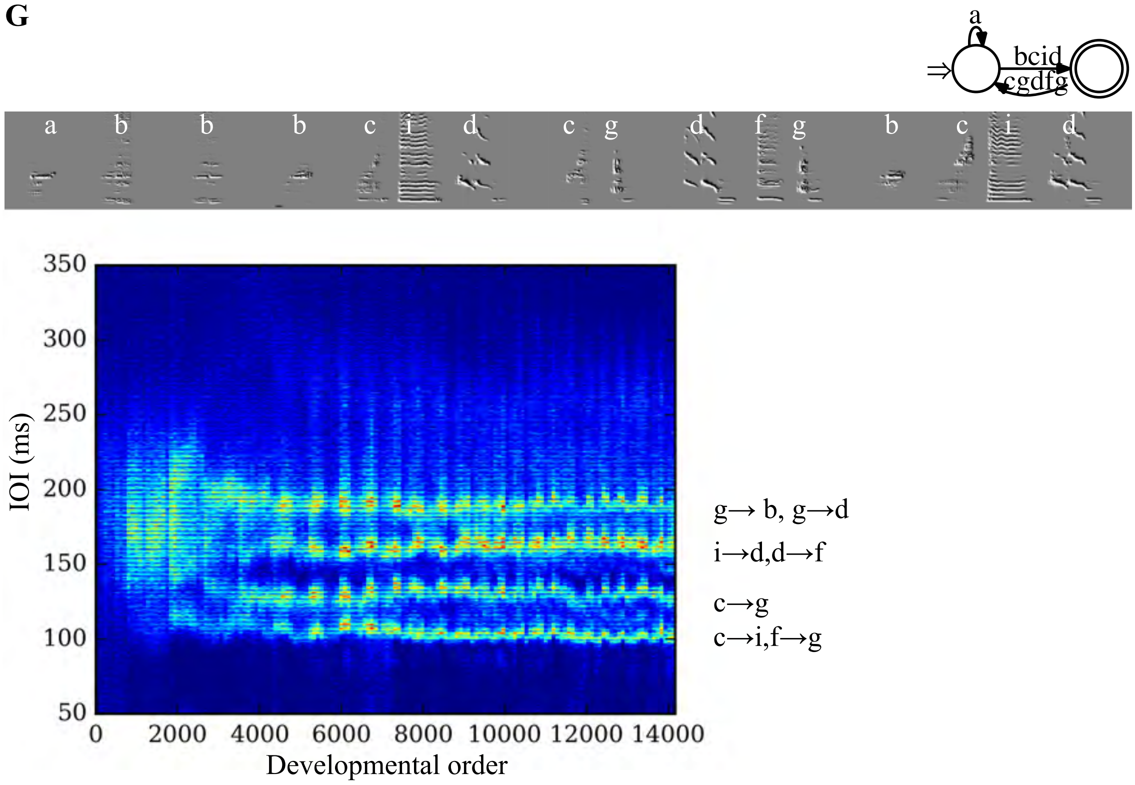

H

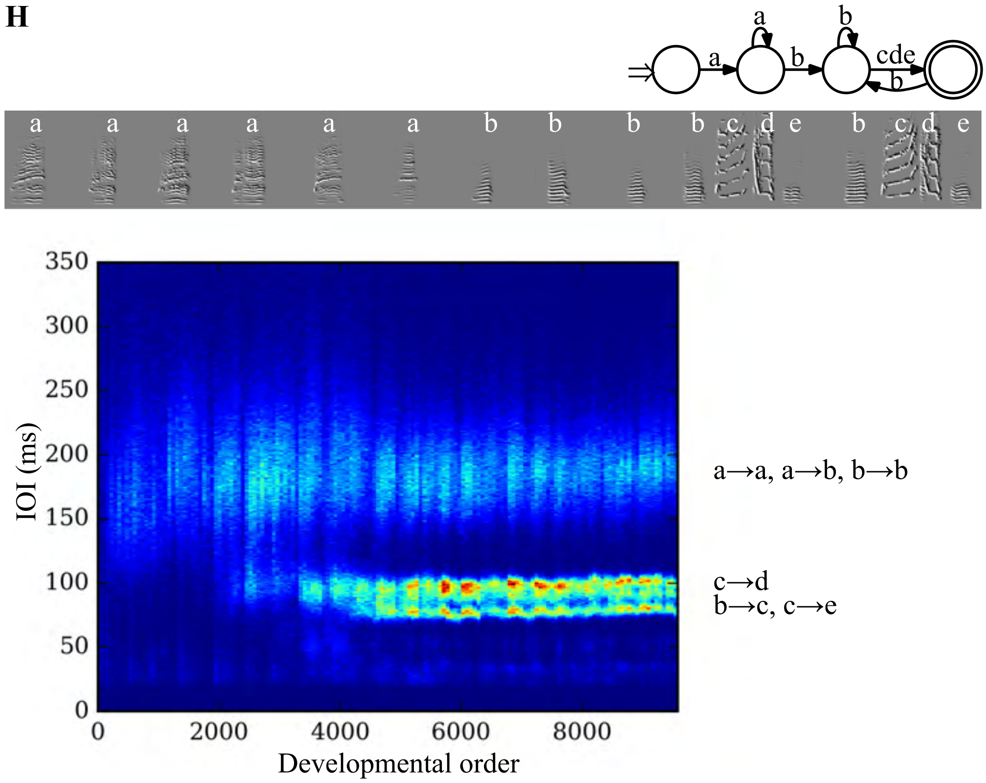

Figure S 7: **Examples of rhythm landscape.** (G) Bird9: # of note types = 8, # of transitions = 20. (H) Bird10: # of note types = 5, # of transitions = 13. Major transitions and transition diagrams are shown.

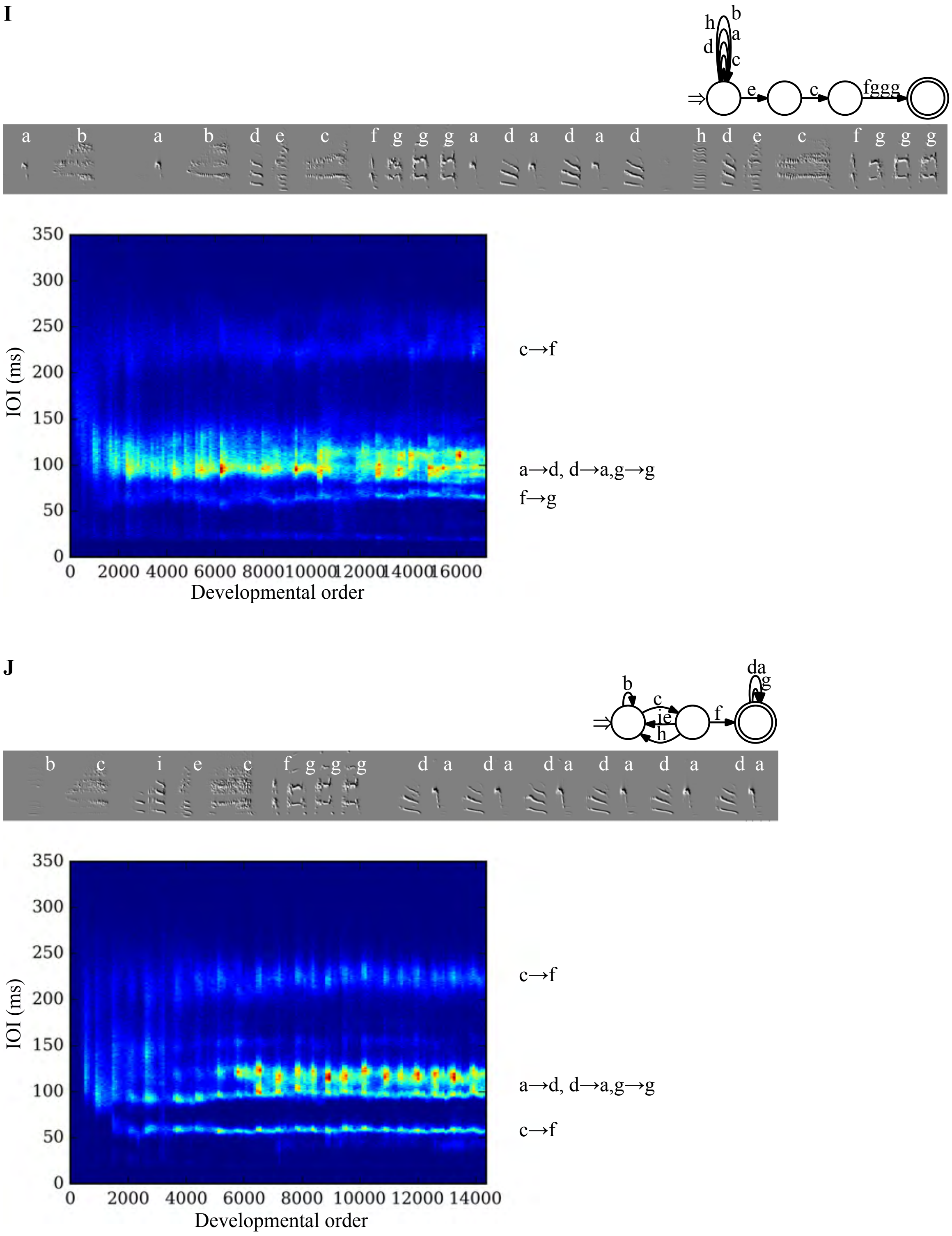

Figure S 8: **Examples of rhythm landscape.** (I) Bird11: # of note types = 6, # of transitions = 22. (J) Bird12: # of note types = 9, # of transitions = 29. Major transitions and transition diagrams are shown.

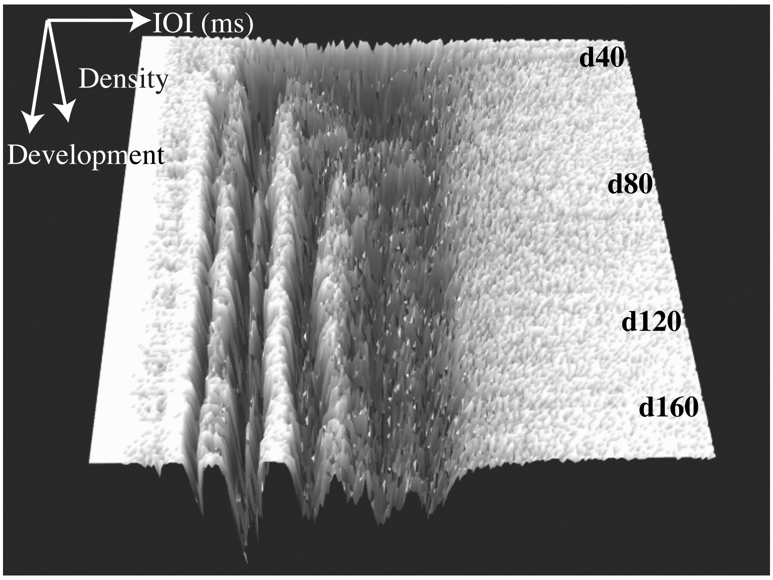

Figure S 9: **3D visualization of the rhythm landscape of a Bengalese finch song (bird2).**

References

[1] Kakishita, Y., Sasahara, K., Nishino, T., Takahasi, M. & Okanoya, K., 2009 Ethological data mining: an automata-based approach to extract behavioral units and rules. *Data Min. Knowl. Disc.* **18**, 446–471. (doi: 10.1007/s10618-008-0122-1).
